# A Turkish translation and validation of the Sense of Agency Scale (SoAS-TR)

**DOI:** 10.3389/fpsyg.2025.1696418

**Published:** 2026-01-12

**Authors:** Işık Batuhan Çakmak, Şeyma Uygun, İrem Kar, Erol Göka

**Affiliations:** 1 Department of Psychiatry, Ankara Bilkent City Hospital, University of Health Sciences, Ankara, Türkiye; 2 High-Security Forensic Psychiatry Unit, Ankara Bilkent City Hospital, Ankara, Türkiye; 3 Department of Biostatistics, Faculty of Medicine, Ankara University, Ankara, Türkiye

**Keywords:** general agency beliefs, questionnaire validation, Sense of Agency (SoA), test–retest reliability, Turkish translation, self-agency, judgment of agency

## Abstract

The sense of agency is defined as the experience of being the initiator of one’s actions and of influencing one’s surroundings. It represents a fundamental aspect of action monitoring, self-recognition, and the ability to distinguish one’s own actions from external events. Disturbances in the sense of agency have been reported across various psychiatric conditions, underscoring the need for valid self-report tools. However, no validated measure has been available to assess general agency beliefs in Turkish. This study aimed to adapt the Sense of Agency Scale into Turkish and evaluate its psychometric properties in an adult community sample. A total of 316 participants (65.5% women; mean age = 36.0, SD = 12.2) completed the survey, and 85 completed a 2-week retest. Confirmatory factor analyses supported the original two-factor structure—Sense of Positive Agency and Sense of Negative Agency—demonstrating good model fit, strong internal consistency, and moderate test–retest reliability. Construct validity was supported through associations with relevant constructs measured by the General Self-Efficacy Scale, the Rotter Internal–External Locus of Control Scale, the Multidimensional Assessment of Interoceptive Awareness, and the Free Will and Determinism Scale. Incremental validity analyses showed that the sense of negative agency explained significant additional variance in depressive symptoms, obsessive-compulsive traits, and schizotypal personality features beyond self-efficacy and locus of control, as assessed by the Beck Depression Inventory, the Obsessive-Compulsive Inventory–Revised, and the Schizotypal Personality Questionnaire, respectively. Associations with the Religiosity Scale were also examined to explore cultural dimensions of agency beliefs. Overall, the findings indicate that the Turkish adaptation is a reliable and valid instrument for assessing general agency beliefs across clinical and non-clinical contexts.

## Introduction

The sense of agency (SoA) refers to the attribution of agency to oneself, i.e., the ability to initiate actions, control them, and change the surrounding world through them (Haggard and Chambon, 2012; Haggard, 2017). Through the SoA, a person is aware of the action he is doing and can therefore differentiate himself as the one doing the action from the other who is not (Balconi, 2010). It is also thought that the sense of agency is related to the subjective experience of the agent rather than to a single, common structure reflecting objective reality. And for any individual, there may be different levels of awareness of both the action itself and its outcome (Buhrmann and Di Paolo, 2017; Moore, 2016).

To date, the sense of agency has been evaluated and defined under various headings (Gallagher, 2007; Synofzik et al., 2008a). In 2008, Synofzik proposed defining SoA under two main headings: the *feeling of agency* and the *judgment of agency* (Synofzik et al., 2008a). The feeling of agency refers to a non-conceptual SoA in which the agent is not explicitly aware of her agency; rather, she focuses on the outcome of the action rather than on being its author. Judgment of agency is defined as an SoA in which a person is aware of her responsibility for her action and explicitly monitors her action processes (Buhrmann and Di Paolo, 2017; Synofzik et al., 2008a). The feeling of agency is more related to sensorimotor processes, whereas judgment of agency seems to be related to causal attribution and beliefs about action (Moore, 2016).

While a sense of agency seems closely related to general well-being, it is reported to be impaired in many psychiatric disorders (Moccia et al., 2024). Studies have reported that positive symptoms in schizophrenia patients may be associated with changes in the agency perception, prediction, awareness, and attribution of action and its outcome (Moccia et al., 2024; Shergill et al., 2005). In patients with depressive disorder, the sense of control may decrease as symptom severity increases, and this may alter agency (Vogel et al., 2024). On the other hand, it has been suggested that patients with obsessive-compulsive disorder may feel an exaggerated level of responsibility and control that is incompatible with objective reality, or, on the contrary, their autonomy over their actions may be significantly reduced (Borrelli et al., 2024). In borderline personality disorder, there may be an abnormality in the sense of ownership of action due to impulse control problems (Moccia et al., 2024; Löffler et al., 2020), and in autism spectrum disorders, there may be problems in the stages of processing sensorimotor signals, planning the action, and evaluating the response (Moccia et al., 2024; Zalla and Sperduti, 2015). Given that disturbances in agency occur across a wide range of psychiatric and neurodevelopmental conditions—and that these alterations involve subjective, phenomenological aspects not fully captured by behavioral or neurobiological measures—there is a clear need for a reliable self-report instrument to assess general agency beliefs in both clinical and experimental contexts.

Some studies have reported slightly higher agentic tendencies in men (Chen et al., 2017), whereas others have found no gender differences (Kalender et al., 2020), and such inconsistencies likely reflect the varied ways in which agency has been conceptualized and measured. Existing research spans minimal, experimentally induced, and self-representational forms of agency, making reported gender effects highly context dependent and generally small in magnitude. Accordingly, it remains uncertain whether these findings extend to the phenomenological dimensions of agency.

To date, the SoA has been measured by direct/explicit and indirect/implicit methods by many researchers (Dewey and Knoblich, 2014; Oren and Eitam, 2017). For indirect measurement, methods such as *intentional binding* and *sensorimotor attenuation* have been used (Haggard et al., 2002; Waszak et al., 2012). Intentional binding was defined as a shorter perception of the time between the voluntary action initiated by the actor and the outcome of the action (Haggard et al., 2002). Sensorimotor attenuation refers to the perception of the outcomes of voluntary actions with less intensity than those of automatic actions. It is related to the prediction of the outcome of the voluntary action prior to the initiation (Waszak et al., 2012; Han et al., 2021). Both methods have been used to measure the SoA in many studies so far (Han et al., 2021; Gu et al., 2025; Moore and Obhi, 2012).

To directly measure the SoA, self-assessment scales and questionnaires were administered to participants, and direct questions were asked about the judgment of the action and the causal relationship (Sato and Yasuda, 2005; Dewey and Carr, 2013). Direct methods were used to measure the explicit attribution and the level of control in the context of the experiment applied during the study (Gentsch et al., 2012). However, researchers have noted that results obtained using indirect and direct methods may not be correlated and suggested that these methods may provide information about distinct mental processes that constitute the sense of agency and have criticized the measurement methods for these reasons (Dewey and Knoblich, 2014).

To measure a general sense of agency independent of the concept and experimental conditions, the Sense of Agency Scale (SoAS) was developed by Tapal et al. (2017). The scale was constructed with two dimensions, “sense of positive agency (SoPA)” and “sense of negative agency (SoNA).” While the SoPA sub-dimension of the scale provides information about the presence or severity of a sense of agency, the SoNA sub-dimension has been reported to indicate not only a lack of agency but also an *existential helplessness*. It was thought that the general self-efficacy belief that the person can create purposeful behaviors and adapt to the changes occurring in the environment and the evaluation of feelings of control over their actions, concepts such as determinism and free will, as well as body monitoring, may be important for a *chronic sense of agency*, so the sense of control, general and physical self-efficacy, free will, and body awareness were evaluated by the research team with scales (Tapal et al., 2017).

In the Turkish literature, the only self-report tool in this area is the adaptation of the Multi-Measure Agentic Personality Scale (MAPS), which assesses agentic functioning through constructs such as self-esteem, purpose in life, internal locus of control, and self-efficacy (Atak et al., 2013). However, MAPS assesses these broader personality-based characteristics rather than the subjective, phenomenological experience of initiating and controlling one’s own actions. The SoAS, by contrast, focuses directly on this experiential aspect of agency and distinguishes between positive and negative forms of perceived control, offering greater sensitivity to subtle variations in subjective agency. Introducing the SoAS into Turkish, therefore, fills an important conceptual gap and provides researchers with a tool specifically designed to assess the phenomenological sense of agency.

## Materials and methods

For the validity and reliability study of the scale in Turkish, permission was obtained from Tapal et al. In our study, the English version of the scale was used to translate into Turkish. The 13 items of the scale were translated into Turkish by two researchers from the research team who were proficient in both Turkish and English. Additionally, two linguists, a philosopher, and a researcher in phenomenology, independent of the research team, were asked to translate the items into Turkish, and both translations were subsequently compared. The 13-item Turkish scale, finalized following the comparison, was translated back into English by another translator who was blind to the original English version using the back-translation approach (Bartram et al., 2018). The original English scale and the retranslated English scale were compared, and the items were evaluated one by one to arrive at the final version of the Turkish 13-item scale. The Turkish translation of the scale has 13 items across two sub-dimensions: six items within the scope of SoPA and seven within the scope of SoNA, in accordance with the original version created by Tapal et al. In each item, a 7-point Likert-type response can be given, ranging from “completely agree” to “completely disagree” (Tapal et al., 2017).

We included the Turkish versions of the General Self-Efficacy Scale, Rotter Internal External Control Scale, Multidimensional Assessment of Introceptive Awareness Scale, and the Free Will and Determinism Scale for the construct validity; the Beck Depression Scale, Schizotypal Personality Questionnaire, and the Obsessive Compulsive Inventory – Revised for incremental validity; and the Religiosity Scale to assess the cultural/religious aspects of SoA.

The study was designed in two stages, with a 2-week interval between them. For the study, an online form created through Google Surveys was used. The target sample consisted of healthy adults aged 18–65 years who were literate. At the beginning of the online survey, participants were informed about the purpose of the study, the eligibility criteria, the structure of the included scales, and the approximate completion time. To ensure data completeness, all items had to be answered before proceeding. Because the survey included multiple scales and was administered online—often via mobile phones—we implemented several procedures to minimize inattentive or careless responding. First, long-string analysis was applied, and participants who provided the same response option across 15 consecutive items were flagged and excluded. Second, two instructed-response attention-check items (e.g., “Choose the one with the largest number from the options.”) were embedded within the SoAS-TR to identify careless responding. Participants who failed either item were removed from the dataset. To reduce monotony, given the number of forms included, each scale was presented on a separate page, with clear headings and formatting distinctions (e.g., bolded titles and spacing) to visually separate it from the previous one. The survey was pilot-tested on both computers and mobile phones to ensure readability and ease of use across devices.

The participants were reached through social media and messaging applications. At the end of the first stage, participants were asked whether they agreed to participate in the second stage. Contact information for participants who agreed to participate was obtained, and they were contacted 2 weeks later. In the second stage, the participants were asked to respond only to the questionnaire containing the Turkish-SoA scale. The decision to use a 2-week retest interval was made to capture potential short-term variability while still reflecting trait-like agency beliefs, ensuring a balance between ecological validity and temporal stability (Koo and Li, 2016).

A total of 370 individuals participated in the first stage of the study; 105 accepted participation in the second stage, and 91 of those completed the second-stage questionnaire. Finally, after excluding participants who responded incorrectly to questions independent of the scale, the data from 316 participants in the first stage and 85 participants in the second stage were evaluated.

### The General Self-Efficacy Scale (GSES)

The GSES was developed by Schwarzer et al. (1995). In 2010, it was translated into Turkish by Aypay, and in the 10-item version used in our study, the items are in a 4-point Likert-type format, rated from “1 = completely false” to “4 = completely true” (Aypay, 2010). In the Turkish version of the scale, the overall Cronbach’s alpha coefficient was found to be 0.83. We examined the relationship between self-efficacy and sense of agency with this scale.

### Locus of Control Scale (LoCS)

The LoCS, developed by Rotter (1966), was translated into Turkish by Dağ (1991), and the 29-item, 2-option version was used in our study. Points are assigned to items according to the a or b options, and a total score between 0 and 23 is calculated. For the Turkish version, the Cronbach’s alpha coefficient was 0.70. The scale is designed to evaluate individuals’ control expectations.

### Multidimensional Assessment of Introceptive Awareness Scale (MAIA-II)

The MAIA-II was developed by Mehling et al. (2018), and a 2nd version, with 37 items, was later published. The second version was unidirectionally translated into Turkish by Munguldar et al. (2018). The scale consists of 8 sub-dimensions of *noticing, not-distracting, not-worrying, attention regulation, emotional awareness, self-regulation, body listening,* and *trusting*. Each item in the scale is rated on a Likert scale from 0 (never) to 5 (always). There are reverse-scored items, and the scale score is calculated by taking the average of the items determined for each sub-dimension. Cronbach’s alpha values for the sub-dimensions in the original MAIA-2 were measured between 0.64 and 0.83. In our study, we planned to evaluate the relationship between bodily awareness and changes in body perception with the sense of agency using the scale.

### Free Will and Determinism Scale (FAD-Plus)

The FAD-Plus was developed by Paulhus and Carey (2011) and comprises 4 sub-dimensions with 27 items; the sub-dimensions are named Free Will, Fatalistic Determinism, Scientific Determinism, and Unpredictability. The scale was adopted in Turkish by Alper and Sümer (2017), and the 3rd item was removed from the Turkish version. The Turkish scale has 26 items; each item is answered on a 5-point Likert scale ranging from “1 = strongly disagree” to “5 = strongly agree.” The Cronbach alpha coefficient for the sub-dimensions of fatalistic determinism was found to be 0.87, for scientific determinism 0.62, for free will 0.66, and for unpredictability 0.80. In our study, we planned to examine the effect of cultural transmission and beliefs on people’s sense of agency with this scale.

### Beck Depression Inventory (BDI)

The scale was originally developed by Beck (1961), and the Turkish validity and reliability study of the scale was conducted by Hisli (1989). In the 21-item scale, each item is scored from 0 to 3, and the total score indicates the severity of depressive symptoms. The Cronbach’s alpha coefficient was found to be 0.80. The presence of subclinical depressive symptoms was evaluated with the BDI in our study.

### Schizotypal Personality Questionnaire (SPQ)

The scale was originally developed by Raine (1991), and its Turkish validity and reliability were examined by Şener et al. (2006). For the 74-item scale, Raine proposed a dual-factor structure as positive schizotypy and negative schizotypy and a triple-factor structure as cognitive-perceptual schizotypy, interpersonal schizotypy, and disorganized schizotypy. Scale questions are scored as yes = 1, no = 2. In the Turkish version, the Cronbach’s alpha coefficient for the scale was 0.91, while the subscales ranged from 0.66 to 0.83. The scale was included in our study to evaluate the relationship between schizotypy dimensions and SoA.

### Obsessive Compulsive Inventory-Revised (OCI-R)

The OCI-R was created in 2002 by Foa et al. (2002), and the Turkish validity and reliability study of the 18-item inventory was conducted by Yorulmaz et al. (2015). The inventory has six sub-dimensions: washing, checking, obsessing, neutralizing, ordering, and hoarding, and its items are on a 5-point Likert scale with “0 = not at all” and “4 = extremely.” While the internal consistency for the total scale in the Turkish OCI-R was 0.9, it ranged from 0.64 to 0.84 across the sub-dimensions. The OCI-R was included in our study to evaluate obsessive-compulsive symptoms and their effect on SoA.

### Religiosity Scale (RS)

The scale developed by Ayten comprises nine questions and two sub-dimensions: faith-influence and knowledge-ritual (Ayten, 2009). In the faith-influence sub-dimension, the scale questions were graded between “1 = not at all effective” and “3 = very effective,” and the effect of religious beliefs on the choices of the individuals was evaluated with these questions, while in the knowledge-ritual dimension, the frequency of religious worship was asked, and the answers were graded between “1 = never” and “3 = always.” Cronbach’s alpha values were 0.80 for the overall scale, 0.743 for the faith-influence sub-dimension, and 0.742 for the knowledge-ritual sub-dimension. In our study, the effect of the participants’ religious beliefs and worship on their sense of agency was evaluated with this scale.

### Statistical analyses

All analyses were conducted in R (version 4.5.0) (R Core Team, 2025). Confirmatory factor analysis (CFA) was performed using the *lavaan* package (Rosseel, 2012). Internal consistency indices (Cronbach’s *α* and McDonald’s *ω*) and zero-order correlations were computed using the *psych* package (Revelle, 2025). Intraclass correlation coefficients (ICC) for test–retest reliability were estimated using the *irr* package (Gamer and Lemon, 2019). Hierarchical regression models and diagnostic checks were conducted in base R, using the *broom* (Robinson et al., 2019) and *car* (Fox and Weisberg, 2019) packages. Partial Spearman correlations were computed using the *ppcor* package (Kim, 2015).

Descriptive statistics were reported as frequencies and percentages for categorical variables and as means with standard deviations for continuous variables. CFA was performed using the lavaan package with the Weighted Least Squares Mean and Variance Adjusted (WLSMV) estimator, which is suitable for ordinal data (Muthén, 1998). Model fit was evaluated using multiple indices: the Comparative Fit Index (CFI), the Tucker-Lewis Index (TLI), the Root Mean Square Error of Approximation (RMSEA), and the Standardized Root Mean Square Residual (SRMR). CFA model fit was interpreted using established thresholds (CFI ≥ 0.90, TLI ≥ 0.90, RMSEA ≤ 0.08, SRMR ≤ 0.08), which indicate acceptable model fit. The factor structure was defined based on the original development study of the scale. Weighted subscale scores were calculated by multiplying each item by its standardized factor loading from the CFA and summing the products to better reflect the latent construct.

Spearman rank-order correlations were used to examine the relationships between the SoAS subscales and external psychological constructs, including scores of LoCS, MAIA-II, FAD-Plus, GSES, BDI, OCI-R, and SPQ. Additionally, partial Spearman correlations were computed between BDI and SoPA/SoNA, controlling for LoCS (external locus of control), GSES, and OCI-R, and between OCI-R and SoPA/SoNA, controlling for LoCS, GSES, and BDI. Internal consistency reliability was assessed using both Cronbach’s alpha and McDonald’s omega coefficients for each subscale. Test–retest reliability was evaluated using the intraclass correlation coefficient (ICC) with a two-way mixed-effects model and absolute agreement type. ICC values were interpreted according to established guidelines (ICC < 0.50 = poor, 0.50–0.75 = moderate, 0.75–0.90 = good, and > 0.90 = excellent reliability).

We tested incremental validity with hierarchical OLS regressions using standardized variables (z-scores). Step 1 included LoCS and GSES; Step 2 added the SoAS subscales: SoPA and SoNA. For each outcome (OCI-R, BDI, SPQ totals), we report standardized coefficients (*β*) with SE(β), t, and p, and we present Adjusted R^2^ once per step. Model improvement (Step 2 vs. Step 1) was evaluated with F-change tests (reported in text). Assumptions were checked via residual diagnostics (linearity, homoscedasticity, and normality), and multicollinearity was assessed using VIF (Variance Inflation Factor); all VIFs were low (≈1.2–1.3), indicating no collinearity concerns. Known-groups validity was examined by testing associations of SoPA and SoNA with age and income using Spearman rank-order correlations and by comparing SoPA/SoNA across gender and education groups using Mann–Whitney U tests; for group comparisons, medians with interquartile ranges (Q1–Q3) were reported.

### Subscale scoring approach

To better reflect the underlying factor structure revealed by the confirmatory factor analysis (CFA), we calculated weighted composite scores for the two SoAS subscales, *SoPA* and *SoNA*. Specifically, each item was multiplied by its standardized factor loading and summed to produce a weighted subscale score. This method provides a construct-valid estimate of participants’ agency experiences, aligning closely with the latent structure supported by the CFA results.

## Results

A total of 316 participants were included in the study. Table 1 presents the sociodemographic and clinical characteristics of the sample. The majority of the participants identified as women (65.5%), with a mean age of 36.0 years (SD = 12.2). The majority of the participants were married, had completed an undergraduate degree, and reported no history of psychiatric or chronic medical conditions.

**Table 1:** Sociodemographic and clinical characteristics of the sample.

Characteristic	*n* = 316
Sex	
Female	207 (65.51%)
Male	109 (34.49%)
Age, *years*	35.98 (12.22)
Marital status	
Single	131 (43.36%)
Married	169 (53.48%)
Divorced	10 (3.16%)
Education level	
Primary school	4 (1.27%)
Secondary school	6 (1.90%)
High school	53 (16.77%)
College/University	253 (80.06%)
Employment status	
Employed	216 (68.35%)
Unemployed	46 (14.56%)
Retired	20 (6.33%)
Student	34 (10.76%)
Income level	
Low	14 (4.43%)
Medium	210 (66.46%)
High	92 (29.11%)
Living arrangement	
Alone	59 (18.67%)
With family	246 (77.85%)
With non-family	11 (3.48%)
Psychiatric diagnosis history	
Present	81 (25.63%)
Absent	235 (74.37%)
Psychiatric medication history	
Yes	121 (38.29%)
No	195 (61.71%)
Chronic illness	
Yes	62 (19.62%)
No	254 (80.38%)

Categorical variables are presented as *n* (%); age is presented as Mean (SD).

### Confirmatory factor analysis (CFA)

**Figure 1:**
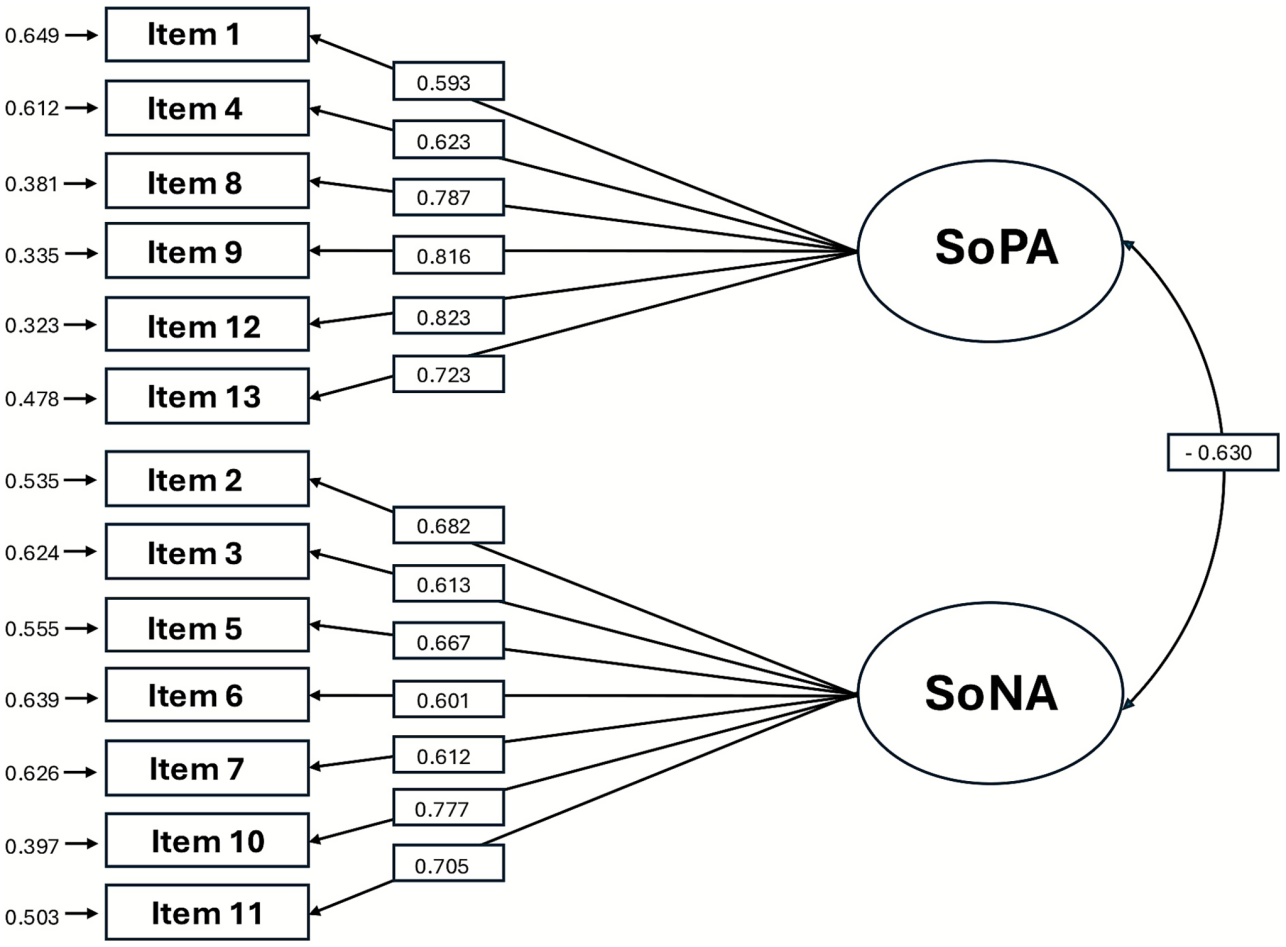
The result of the confirmatory factory analysis.

Confirmatory factor analysis was conducted using the WLSMV estimator due to the ordinal nature of the data. The hypothesized two-factor model, consisting of *SoPA* (Items 1, 4, 8, 9, 12, and 13) and *SoNA* (Items 2, 3, 5, 6, 7, 10, and 11), showed good overall model fit according to commonly recommended criteria [CFI/TLI ≥ 0.90; RMSEA/SRMR ≤ 0.08 (Brown, 2015; Hu and Bentler, 1999): χ^2^(64) = 157.53, *p* < 0.001; CFI = 0.972; TLI = 0.966; RMSEA = 0.068 (90% CI, 0.055–0.082); SRMR = 0.053]. Standardized factor loadings ranged from 0.593 to 0.823 for *SoPA* items and from 0.601 to 0.777 for *SoNA* items (Table 2). All loadings were statistically significant (p < 0.001). The two latent factors were moderately and negatively correlated (r = −0.63, *p* < 0.001), consistent with theoretical expectations. Residual variance values ranged from 0.323 to 0.649, indicating moderate to high proportions of explained variance (Figure 1). Skewness and kurtosis values for each CFA item are provided in Supplementary Table 2.

**Table 2:** Standardized factor loadings from the two-factor CFA model for SoAS-TR.

Item	SoPA	SoNA	*p*-value
Item 1	0.593 (0.039)	–	<0.001
Item 4	0.623 (0.038)	–	<0.001
Item 8	0.787 (0.027)	–	<0.001
Item 9	0.816 (0.026)	–	<0.001
Item 12	0.823 (0.026)	–	<0.001
Item 13	0.723 (0.030)	–	<0.001
Item 2	–	0.682 (0.043)	<0.001
Item 3	–	0.613 (0.041)	<0.001
Item 5	–	0.667 (0.040)	<0.001
Item 6	–	0.601 (0.046)	<0.001
Item 7	–	0.612 (0.044)	<0.001
Item 10	–	0.777 (0.039)	<0.001
Item 11	–	0.705 (0.044)	<0.001

Standardized factor loadings are presented with standard errors in parentheses.

The corrected item–total correlations and Cronbach’s alpha if item deleted values for all SoAS items are presented in Supplementary Table 3. For the SoPA subscale, corrected item–total correlations ranged from 0.441 to 0.685, and removing any item did not improve internal consistency (*α* if deleted = 0.751–0.808). For the SoNA subscale, corrected item–total correlations ranged from 0.440 to 0.522, with α if deleted values between 0.733 and 0.750. These results indicate that all items contributed adequately to their respective subscales and that internal consistency remained stable across item-deletion tests.

### Associations with other psychological measures

Internal consistency coefficients were examined for all external measures included in the study. Reliability estimates were satisfactory across scales. For the RS, internal consistency was high for both Faith & Influence (α = 0.93, *ω* = 0.93) and Knowledge & Ritual (α = 0.89, ω = 0.89). The LoCS demonstrated acceptable reliability (α = 0.70, ω = 0.71). Across the MAIA-II subscales, internal consistency ranged from acceptable to excellent (α = 0.53–0.89; ω = 0.55–0.90). The FAD-Plus subscales also showed adequate reliability (α = 0.66–0.82; ω = 0.69–0.82). The GSES demonstrated strong internal consistency (α = 0.88, ω = 0.89), as did the BDI (α = 0.89, ω = 0.90) and the OCI-R (α = 0.92, ω = 0.92). For the SPQ, internal consistency was high across higher-order factors (α = 0.86–0.95; ω = 0.87–0.95) and for the total score (α = 0.95, ω = 0.95). Because SPQ items are dichotomous, these α values correspond to KR-20 coefficients (see Supplementary Table 1).

Spearman correlation analyses revealed distinct patterns of associations for the two subscales. As presented in Table 3, SoPA demonstrated positive associations with adaptive constructs such as free will beliefs (*r* = 0.455, *p* < 0.001), general self-efficacy (*r* = 0.354, *p* < 0.001), and multiple dimensions of interoceptive awareness, whereas SoNA was positively associated with external locus of control (*r* = 0.381, *p* < 0.001) and negatively associated with free will beliefs (*r* = −0.189, *p* < 0.001). Additionally, SoNA showed statistically significant negative correlations with Noticing (*r* = −0.163, *p* = 0.004), Not-Worrying (*r* = −0.139, *p* = 0.014), Attention Regulation (*r* = −0.172, *p* = 0.002), Emotional Awareness (*r* = −0.118, *p* = 0.036), Self-Regulation (*r* = −0.165, *p* = 0.003), Body Listening (*r* = −0.138, *p* = 0.014), and Trusting (*r* = −0.215, *p* < 0.001). Notably, no significant associations were observed between either SoPA or SoNA and the two dimensions of religiosity (faith and influence; knowledge and ritual).

**Table 3:** Spearman correlations between the subdimensions of the SoAS and other psychological measures.

Scale/Subscale	SoPA		SoNA	
	r	*p*	r	*p*
Construct validity				
RS				
Faith & influence	−0.040	0.480	−0.003	0.955
Knowledge & ritual	−0.095	0.091	0.000	0.994
LoCS – Total score	−0.331	<0.001	0.381	<0.001
MAIA-II				
Noticing	0.267	<0.001	−0.163	0.004
Not-distracting	−0.003	0.952	−0.099	0.080
Not-worrying	0.133	0.018	−0.139	0.014
Attention regulation	0.264	<0.001	−0.172	0.002
Emotional awareness	0.202	<0.001	−0.118	0.036
Self-regulation	0.190	<0.001	−0.165	0.003
Body listening	0.132	0.019	−0.138	0.014
Trusting	0.241	<0.001	−0.215	<0.001
FAD-Plus				
Fatalistic determinism	−0.084	0.136	0.218	<0.001
Scientific determinism	0.101	0.073	0.103	0.068
Free Will	0.455	<0.001	−0.189	<0.001
Unpredictability	0.001	0.987	0.269	<0.001
GSES	0.354	<0.001	−0.243	<0.001
Incremental validity				
BDI – Total score^1^	−0.136	0.016	0.269	<0.001
OCI-R – Total score^1^	−0.055	0.334	0.215	<0.001
SPQ				
Cognitive-Perceptual	−0.146	0.010	0.300	<0.001
Interpersonal	−0.184	0.001	0.397	<0.001
Disorganized	−0.212	<0.001	0.374	<0.001
Positive schizotypy	−0.174	0.002	0.337	<0.001
Negative schizotypy	−0.184	0.001	0.397	<0.001
Total score	−0.193	<0.001	0.400	<0.001

^1^For BDI – Total score, correlations were adjusted for LoCS, GSES, and OCI-R total; for OCI-R – Total score, correlations were adjusted for LoCS, GSES, and BDI – Total score. BDI, Beck Depression Inventory; FAD – Plus, Free Will and Determinism Scale – Plus; GSES, General Self-Efficacy Scale; LoCS, Locus of Control Scale; MAIA-II, Multidimensional Assessment of Interoceptive Awareness – II Scale; OCI-R, Obsessive Compulsive Inventory – Revised; RS, Religiosity Scale; SPQ, Schizotypal Personality Questionnaire; SoPA, sense of positive agency; SoNA, sense of negative agency.

Regarding incremental validity, SoNA showed significant positive correlations with the BDI Total score (*r* = 0.419, *p* < 0.001), the SPQ Total score (*r* = 0.400, *p* < 0.001), and the OCI-R Total score (*r* = 0.318, *p* < 0.001). Given the theoretical overlap among these constructs, partial correlations were calculated to control for potential confounding effects. Specifically, for the BDI Total, correlations were adjusted for LoCS, GSES, and OCI-R Total scores; for the OCI-R Total, correlations were adjusted for LoCS, GSES, and BDI Total scores.

### Incremental validity

Adding the SoAS subscales at Step 2 improved model fit for all outcomes: adjusted R^2^ increased from 0.045 to 0.136 for OCI-R, from 0.142 to 0.249 for BDI, and from 0.104 to 0.220 for SPQ; F-change tests were significant for all three models (*p* < 0.001). In the Step-2 models, SoNA was a consistent positive predictor (OCI-R *β* = 0.330; *p* < 0.001; BDI *β* = 0.335, *p* < 0.001; SPQ *β* = 0.365, *p* < 0.001), whereas SoPA was not significant (OCI-R *β* = −0.025; *p* = 0.673, BDI *β* = −0.073, *p* = 0.190; SPQ *β* = −0.039, *p* = 0.498). LoCS showed small positive associations with OCI-R (*β* = 0.120, *p* = 0.044) and SPQ (*β* = 0.167, *p* = 0.003) but not BDI (*β* = 0.106, *p* = 0.057). General self-efficacy was positively related to OCI-R (*β* = 0.162, *p* = 0.005), inversely related to BDI (*β* = −0.172, *p* = 0.002), and unrelated to SPQ (*β* = −0.018, *p* = 0.746). Collectively, these results indicate that SoNA provides incremental explanatory value beyond control beliefs and self-efficacy, whereas SoPA does not (Table 4).

**Table 4:** Hierarchical regression predicting OCI-R, BDI-II, and SPQ total scores.

Model/Predictor	β	SE(β)	t	*p* value	Adj. R^2^
OCI-R					
Step 1					0.045
LoCS	0.236	0.058	4.040	<0.001	
GSES	0.113	0.058	1.926	0.055	
Step 2					0.136
LoCS	0.120	0.059	2.026	0.044	
GSES	0.162	0.058	2.814	0.005	
SoPA	−0.025	0.060	−0.423	0.673	
SoNA	0.330	0.059	5.543	<0.001	
BDI					
Step 1					0.142
LoCS	0.235	0.055	4.246	<0.001	
GSES	−0.235	0.055	−4.236	<0.001	
Step 2					0.249
LoCS	0.106	0.055	1.912	0.057	
GSES	−0.172	0.054	−3.201	0.002	
SoPA	−0.073	0.056	−1.314	0.190	
SoNA	0.335	0.055	6.049	<0.001	
SPQ					
Step 1					0.104
LoCS	0.298	0.057	5.255	<0.001	
GSES	−0.075	0.057	−1.332	0.184	
Step 2					0.220
LoCS	0.167	0.056	2.955	0.003	
GSES	−0.018	0.055	−0.324	0.746	
SoPA	−0.039	0.057	−0.678	0.498	
SoNA	0.365	0.057	6.459	<0.001	

BDI, Beck Depression Inventory; GSES, General Self-Efficacy Scale; LoCS, Locus of Control Scale; OCI-R, Obsessive Compulsive Inventory – Revised; SE, standard error; SPQ, Schizotypal Personality Questionnaire; SoPA, sense of positive agency; SoNA, sense of negative agency.

### Known-groups validity

Known-groups validity was evaluated via correlations with age and income and comparisons by gender and education. Spearman correlations showed no associations with age or income (SoPA-Age r_s_ = 0.043, *p* = 0.451; SoNA-Age r_s_ = −0.083, *p* = 0.141; SoPA-Income r_s_ = −0.087, *p* = 0.124; SoNA-Income r_s_ = 0.014, *p* = 0.799). By education primary/secondary (*n* = 63) versus college/university (*n* = 253), SoPA was higher in the college/university group [26.16 (22.39–28.88) vs. 24.99 (19.31–27.22), *p* = 0.024], and SoNA was lower [7.93 (5.44–10.64) vs. 10.67 (8.00–16.15), *p* < 0.001].

In terms of gender, men and women did not differ significantly in BDI, OCI-R, or SPQ total scores. Mann–Whitney U tests indicated that women scored higher than men on the Religiosity–Knowledge & Ritual subscale [median (Q1–Q3) = 10.00 (7.00–13.00) vs. 8.00 (5.00–12.00), *p* = 0.011], whereas no significant difference emerged for the Faith & Influence subscale [13.00 (10.00–15.00) vs. 12.00 (7.00–15.00), *p* = 0.159]. Weighted SoPA scores were higher in men compared to women [26.98 (23.21–29.70) vs. 25.37 (21.62–27.75), *p* = 0.004], whereas weighted SoNA scores did not differ between genders [7.71 (5.44–11.06) vs. 8.64 (5.91–11.73), *p* = 0.268]. In an unadjusted linear model, male sex was associated with higher SoPA scores [B = 1.19, 95% CI (0.04, 2.33), *p* = 0.042]. However, after adjusting for the Religiosity–Knowledge & Ritual subscale, this effect decreased and was no longer statistically significant [*B* = 1.03, 95% CI (−0.12, 2.18), *p* = 0.080].

### Reliability Analysis

The reliability analyses for the SoAS-TR are shown in Table 5.

**Table 5:** Reliability analyses for the sense of agency scale.

Subscale	Cronbach’s Alpha (α)	McDonald’s Omega (ω)
Positive agency	0.808	0.815
Negative agency	0.769	0.771

The test–retest subsample consisted of 85 participants. The mean age was 35.1 years (SD = 12.1). Education was presented both continuously and categorically; participants had an average of 15.3 years of schooling (SD = 1.73), and most held a college/university degree (85.9%), followed by high school (11.8%) and secondary school (2.4%). The gender distribution was 56.5% women and 43.5% men. Regarding employment status, 68.2% were employed, 15.3% were students, 11.8% were unemployed, and 4.7% were retired. Test–retest reliability of SoPA and SoNA was assessed using single-measure, two-way mixed-effects intraclass correlation coefficients (ICC) with absolute agreement. The results indicated moderate reliability for both subscales: ICC = 0.614 (95% CI, 0.463–0.731, *p* < 0.001) for SoPA and ICC = 0.705 (95% CI, 0.580–0.798, *p* < 0.001) for SoNA.

## Discussion

This study aimed to provide a validated Turkish version of the SoAS. The two-factor structure of the SoAS-TR, comprising the SoPA and the SoNA, was strongly supported by CFA in our sample, with excellent fit indices and substantial item-level loadings. This finding replicates the original validation (Tapal et al., 2017) and aligns with the French (Hurault et al., 2020), German (Bart et al., 2023), and Japanese (Xu et al., 2024) adaptations, all of which demonstrated a consistent two-factor structure reflecting adaptive and maladaptive aspects of agency beliefs.

The moderate negative correlation between SoPA and SoNA in our sample is consistent with theoretical expectations that positive and negative agency are not opposite poles of a single dimension but rather distinct yet interrelated constructs. Tapal et al. (2017) originally reported a weaker correlation, suggesting greater independence between the two dimensions in the original Hebrew sample. By contrast, the German and French validations yielded stronger inverse associations, which are more aligned with our findings. This variability across studies may reflect differences in sample characteristics or cultural attitudes toward personal control.

In our sample, age was not significantly associated with either SoPA or SoNA, indicating that agency-related beliefs remained relatively stable across the adult age range represented in this study. By contrast, the Japanese validation study examined age subgroups in more detail and found results that aligned with those of Tapal et al. (2017) and others, but were closer to those of Bart et al. (2023) (Xu et al., 2024). The relatively higher mean age of our sample compared to the other cohorts may partially explain the similarity between our findings and those of the other studies. Nevertheless, research that explicitly examines the role of cultural and societal structures will be essential to better understand how age and agency interact across different populations.

Although our sample did not reveal significant differences in agency levels across socioeconomic strata, several considerations suggest that socioeconomic status (SES) may still play a meaningful role in shaping beliefs about agency. Higher SES is often associated with greater autonomy, access to resources, and opportunities for self-directed action, which can foster stronger beliefs in personal control and efficacy. In contrast, lower SES has been linked to increased sensitivity to contextual constraints and external influences, potentially reinforcing a more externalized sense of agency (Miyamoto and Ji, 2011). Moreover, agency-related constructs, such as self-concept and perceived control, have been shown to partially mediate the effects of SES on educational and occupational outcomes (Parker et al., 2012), supporting the notion that agency may serve as a psychological pathway through which SES influences life trajectories. Given that most participants in our sample were university graduates, employed, from medium- to high-income groups, and lived with their families, the sample largely represents individuals who are self-sufficient and capable of assuming various responsibilities in daily life. Factors that increase personal efficacy are known to support individuals in enacting their agency and transforming adverse life experiences into actions that benefit themselves and their communities. Therefore, a sample representing a broader range of SES levels would likely yield more accurate and generalizable results.

Confirmatory factor analysis further supported the two-factor structure, and the careful, multidisciplinary translation team ensured that the original 13 items were preserved in the Turkish version, highlighting the importance of careful cultural and linguistic adaptation to maintain structural fidelity. Internal consistency was good for both subscales, with Cronbach’s *α* and McDonald’s *ω* values exceeding 0.77, replicating the reliability levels observed in the original and other adaptations.

The pattern of associations between SoAS-TR subscales and external psychological measures in our sample provides evidence of construct validity and offers deeper insights into the mechanisms underlying positive and negative agency. Consistent with theoretical models of agency and prior validations (Tapal et al., 2017; Bart et al., 2023; Xu et al., 2024), SoPA showed strong positive correlations with free will beliefs and general self-efficacy, suggesting that individuals who perceive themselves as effective agents tend to hold stronger beliefs in personal autonomy and competence. This aligns with hierarchical and predictive coding models of agency, which posit that a stable sense of authorship emerges when internal predictions about action-outcome contingencies are reinforced by successful interactions with the environment (Synofzik et al., 2008b).

Importantly, SoPA was also positively associated with multiple dimensions of interoceptive awareness, including attention regulation, emotional awareness, and body trust. These findings converge with neurocognitive and embodied accounts of agency, which highlight the role of integrating bodily signals into higher-order cognitive models of control (Balconi, 2010). The positive links between SoPA and adaptive bodily awareness support the idea that enhanced integration of sensorimotor and interoceptive cues fosters a coherent and stable sense of agency (Bart et al., 2023). Similar results were noted in the German and Japanese validations, suggesting that the embodiment of agency may be a cross-culturally robust feature of SoPA (Bart et al., 2023; Xu et al., 2024). Conversely, SoNA exhibited the opposite pattern, showing positive associations with an external locus of control and negative associations with interoceptive and regulatory capacities, such as body trust, attention regulation, and self-regulation. This pattern underscores SoNA as reflecting vulnerability to the disrupted integration of internal signals into the sense of control, consistent with frameworks linking SoNA to helplessness, over-reliance on external attributions, and reduced sensorimotor confidence (Tapal et al., 2017; Hurault et al., 2020).

Taken together, these findings highlight that SoPA and SoNA represent distinct but complementary dimensions of general agency beliefs: SoPA as an adaptive dimension characterized by internal control and embodied coherence, and SoNA as a maladaptive dimension reflecting externalized control and attenuated integration of bodily and emotional information. This multidimensionality appears to be culturally stable, as demonstrated in the original, German, Japanese, and French adaptations, though subtle cultural variations may shape the strength and expression of these associations (Tapal et al., 2017; Hurault et al., 2020; Bart et al., 2023; Xu et al., 2024).

Test–retest reliability over the two-week interval in our study was moderate, consistent with prior adaptations that used longer intervals (Tapal et al., 2017; Bart et al., 2023). The shorter retest duration in our design was chosen to minimize situational influences and to balance stability and ecological validity (Koo and Li, 2016), given that agency beliefs may fluctuate with mood, environmental changes, or acute stressors (Moore and Obhi, 2012; Lawrance et al., 2022). Nonetheless, the moderate stability observed highlights the need for further research into the situational variability of agency beliefs, despite their conceptualization as cross-situational or chronic traits.

The psychopathological relevance of the SoAS-TR is well established, as SoA alterations have been consistently implicated across a wide range of psychiatric and neuropsychiatric conditions, including schizophrenia, OCD, and depression (Oren et al., 2016; Giuliani et al., 2021; Malik et al., 2022; Salgado-Pineda et al., 2022). SoA is increasingly conceptualized as a transdiagnostic vulnerability marker with both trait-like and state-dependent features (Malik et al., 2022). In our study, SoNA showed positive associations with depressive and obsessive-compulsive features. Additionally, SoNA correlated with all dimensions of schizotypy, including cognitive-perceptual, interpersonal, and disorganized subdimensions. This pattern strongly aligns with the growing body of evidence linking diminished agency to psychopathological processes involving learned helplessness, impaired self-monitoring, and externalized control beliefs (Synofzik et al., 2008a; Tapal et al., 2017; Asai and Tanno, 2008; Kozáková et al., 2020).

A recent review by Malik et al. (2022) emphasizes that schizophrenia is perhaps the most thoroughly studied condition with respect to agency disruptions, with robust evidence indicating abnormalities in both low-level predictive processing and high-level self-reflective mechanisms. Neuroimaging studies demonstrate that alterations in the temporoparietal junction and associated cortical networks underlie impaired self-other differentiation and agency attributions in schizophrenia (Salgado-Pineda et al., 2022). These neural mechanisms help explain the pervasive experiences of external control and thought insertion characteristic of positive symptoms, while also linking reduced agency to poor clinical insight (Salgado-Pineda et al., 2022; Amdie et al., 2022).

In OCD, the literature consistently reports that maladaptive agency beliefs—often characterized by an overestimation of responsibility and distorted control beliefs—contribute to symptom severity. For example, Giuliani et al. (2021) note that patients with checking compulsions often report a lack of “action-completion” sensations, reflecting alterations in agency attribution and cognitive inflexibility in tasks that require accurate self–action monitoring (Giuliani et al., 2021). Similarly, Gentsch et al. (2012) showed that individuals with OCD exhibit dysfunctional forward model mechanisms, which impair accurate prediction and monitoring of self-generated actions, leading to a disrupted sense of agency (Gentsch et al., 2012). These findings align with our observation that SoNA is positively related to obsessive traits even when controlling for depressive symptoms. Regarding depression, SoA disturbances are commonly interpreted through the lens of learned helplessness models, where reduced perceptions of control over actions and outcomes perpetuate passive coping and low motivation (Malik et al., 2022). Across all studies, higher SoNA consistently aligns with higher depressive symptoms, reinforcing the link between negative agency and mood pathology (Tapal et al., 2017; Bart et al., 2023; Xu et al., 2024), whereas SoPA is unassociated mainly with depression, suggesting that positive agency does not directly buffer depressive symptoms, or that its protective effect is mediated through other constructs like self-efficacy or locus of control.

Collectively, these results converge on the view that SoNA indexes maladaptive, externally oriented agency beliefs associated with psychopathological vulnerability, whereas SoPA reflects adaptive, internalized control beliefs that support resilience. As Malik et al. (2022) emphasize, integrating both implicit (feeling of agency) and explicit (judgment of agency) measures in future studies could clarify the hierarchical and interactive processes through which agency distortions emerge and manifest across disorders (Malik et al., 2022). This approach may also inform clinical interventions, as enhancing SoA—through cognitive remediation, metacognitive therapy, or agency-focused psychotherapies—has been proposed as a promising strategy to improve treatment adherence, functional outcomes, and recovery trajectories.

The relationship between religiosity and SoA has long been theorized, given that many faith systems shape beliefs about autonomy, responsibility, and control (Spilka et al., 1985; Paylor, 2018; Liu and Froese, 2020). Religiosity, broadly defined, encompasses beliefs, practices, and identifications related to spiritual or theological frameworks, while the SoAS captures general, cross-situational beliefs about agency through its SoPA and SoNA subscales. Prior literature indicates that internalized, adaptive forms of faith — such as beliefs in divine partnership or moral responsibility — can enhance agency and resilience (Latif et al., 2018), whereas fatalistic or deterministic beliefs may undermine perceived control (Fiori et al., 2006). Despite these theoretical connections, our study found no significant associations between religiosity and either SoPA or SoNA. Several explanations may account for this null finding. First, cultural and contextual nuances are critical. In collectivist or secular environments, faith may emphasize communal belonging or obedience over individual control, as shown in studies of Indonesian university students and Vietnamese Catholic families (Liu and Froese, 2020; Nguyen, 2023). Second, religiosity is inherently multidimensional, and aggregate scores may obscure opposing pathways, with some beliefs fostering empowerment and others promoting passivity (Fiori et al., 2006). Third, the SoAS-TR captures chronic, trait-like beliefs, whereas religious frameworks may influence situational or context-dependent experiences of agency (Aarts and van den Bos, 2011). Finally, secularization trends and individual differences in the internalization of faith could attenuate observable associations in non-clinical, heterogeneous samples.

In our study, a significant gender difference in SoPA scores emerged when agency was taken into account. Within the context of Turkey’s heterogeneous yet predominantly collectivistic sociocultural structure, this finding may be understood through the well-established pattern in which gender roles manifest as more communal in women and more agentic in men (Sakallı Uğurlu et al., 2021). Consistent with prior research on Turkish samples, evaluations of gender-related stereotypes indicate that men are associated with power-laden themes—such as dominance, masculinity, and authority—and with social roles such as being the head of the household or a father (Sakallı Uğurlu et al., 2018). Conversely, women are described in terms of personality traits such as warmth and fragility, as well as relational roles such as motherhood, reflecting a strong communal orientation (Sakallı Uğurlu et al., 2018; Sunar, 1982). Moreover, more collectivistic societies may evaluate collectivistic attributes within a masculine framework, and in such contexts, men’s communion can paradoxically manifest through relational leadership and protective roles (Cuddy et al., 2015).

On the other hand, the loss of statistical significance of the gender difference in SoPA scores after adjusting for the Knowledge–Ritual subscale of the RS may reflect an increase in communal tendencies among individuals in societies where religious teachings are deeply embedded in the social fabric (Gebauer et al., 2013). Whereas women’s self-construals tend to manifest within a communal framework shaped by gender role expectations, elevated levels of personal religiosity among men may reflect a community-oriented self-concept that is further reinforced and socialized through engagement in religious rituals (Gebauer et al., 2013).

In our study, a significant difference was found between SoPA and gender, whereas no such difference emerged for SoNA. In the existing literature on gender and agency, SoAS has not been utilized, and commonly used assessment methods have not examined negative forms of agency (Hsu et al., 2021; Gustafsson Sendén et al., 2019). Studies investigating negative and positive masculinity or femininity also do not fully correspond to the type of agency measured by SoNA (Gustafsson Sendén et al., 2019). This aligns with our findings and supports the view that SoPA and SoNA are not simple opposites but instead represent distinct structures related to agency.

This study has several limitations. First, while the sample size was adequate for psychometric analyses, larger and more diverse samples would strengthen generalizability. Additionally, future research may benefit from a preliminary pilot study to assess face validity. Second, the cross-sectional design precludes causal inferences regarding the relationships between agency, psychopathology, and other external measures. Third, the impact of culture on agency was examined primarily through religiosity and basic demographic indicators; future studies should assess broader sociocultural dimensions, such as individualism–collectivism, gender roles, and altruistic values, which may shape beliefs about agency. Fourth, while our study assumed religious homogeneity, the inclusion of participants from diverse faith traditions in future research — ideally in multi-center studies with standardized instruments — would allow for more detailed cross-cultural analyses. Future research should employ more nuanced measures of religiosity to explore whether specific theological beliefs (e.g., free will, divine support, predestination) differentially relate to SoPA and SoNA across diverse cultural settings.

Additionally, although the associations of SoA with psychopathological constructs were examined, our sample did not consist of a clinical population. Therefore, the ways in which SoA may differ between healthy individuals and those with psychiatric disorders remain unclear. Finally, while we explored associations between SoA and self-reported psychopathology, longitudinal and ecological studies are needed to understand the temporal dynamics of agency, its neurocognitive correlates, and its role as a transdiagnostic vulnerability factor. Future studies should also investigate neurocognitive mechanisms underlying agency, bridging psychological and biological perspectives, and evaluate intervention strategies, such as agency-enhancing psychotherapies, for their potential to mitigate psychopathological risk.

## Conclusion

The present study provides a reliable and valid Turkish adaptation of the Sense of Agency Scale (SoAS-TR), supporting its robust two-factor structure and strong psychometric properties. Consistent with previous validations, SoPA and SoNA emerged as distinct yet related dimensions of agency. Future research should further explore cultural, clinical, and neurocognitive factors that influence agency, as well as its role as a potential transdiagnostic marker in psychopathology.

## Data Availability

The raw data supporting the conclusions of this article will be made available by the authors, without undue reservation.
