# Supplementary material for: A Turkish translation and validation of the Sense of Agency Scale (SoAS-TR)
Source: Front Psychol. 2026 Jan 12;16:1696418. doi: 10.3389/fpsyg.2025.1696418 (PMC12832476; doi:10.3389/fpsyg.2025.1696418)
Supplement: Supplementary file 1 [file Table_1.docx]

**Supplementary Table 1: Internal consistency coefficients for the scales and subscales used in the study**

| **Scale** | **Subscale** | **n items** | **α** | **ω** |
| --- | --- | --- | --- | --- |
| RS | Faith & Influence | 5 | 0.93 | 0.932 |
| RS | Knowledge & Ritual | 4 | 0.887 | 0.891 |
| LoCS | Total | 23 | 0.704 | 0.705 |
| MAIA-II | Noticing | 4 | 0.76 | 0.764 |
| MAIA-II | Not-Distracting | 6 | 0.772 | 0.788 |
| MAIA-II | Not-Worrying | 5 | 0.529 | 0.554 |
| MAIA-II | Attention Regulation | 7 | 0.862 | 0.865 |
| MAIA-II | Emotional Awareness | 5 | 0.859 | 0.867 |
| MAIA-II | Self-Regulation | 4 | 0.799 | 0.816 |
| MAIA-II | Body Listening | 3 | 0.889 | 0.890 |
| MAIA-II | Trusting | 3 | 0.862 | 0.868 |
| FAD-Plus | Fatalistic Determinism | 5 | 0.821 | 0.824 |
| FAD-Plus | Scientific Determinism | 7 | 0.663 | 0.686 |
| FAD-Plus | Randomness | 7 | 0.758 | 0.762 |
| FAD-Plus | Free Will | 7 | 0.748 | 0.752 |
| GSES | Total | 10 | 0.884 | 0.887 |
| BDI | Total | 21 | 0.887 | 0.900 |
| OCI-R | Total | 18 | 0.915 | 0.915 |
| SPQ | Cognitive-Perceptual | 33 | 0.913 | 0.918 |
| SPQ | Interpersonal | 34 | 0.911 | 0.914 |
| SPQ | Disorganized | 15 | 0.862 | 0.871 |
| SPQ | Positive Schizotypy | 48 | 0.937 | 0.941 |
| SPQ | Negative Schizotypy | 34 | 0.911 | 0.914 |
| SPQ | Total | 74 | 0.951 | 0.954 |

For dichotomous items, Cronbach’s α corresponds to the KR-20 coefficient.

BDI = Beck Depression Inventory; FAD-Plus: Free Will and Determisim Scale; GSES = General Self Efficacy Scale; LoCS = Locus of Control Scale; MAIA-II = Multidimensional Assessment of Introceptive Awareness Scale ; OCI-R = Obsessive Compulsive Inventory-Revised; RS = Religiosity Scale; SPQ = Schizotypal Personality Questionnaire

**Supplementary Table 2. Item-level distributional properties for the SoAS-TR.**

| **Item** | **n** | **mean** | **sd** | **skewness** | **kurtosis** |
| --- | --- | --- | --- | --- | --- |
| SoAS_1 | 316 | 5.40 | 1.68 | -1.196 | 0.403 |
| SoAS_2 | 316 | 1.75 | 1.28 | 1.723 | 1.835 |
| SoAS_3 | 316 | 2.53 | 1.73 | 0.891 | -0.647 |
| SoAS_4 | 316 | 5.81 | 1.46 | -1.546 | 1.873 |
| SoAS_5 | 316 | 2.02 | 1.51 | 1.522 | 1.204 |
| SoAS_6 | 316 | 2.19 | 1.67 | 1.294 | 0.344 |
| SoAS_7 | 316 | 2.40 | 1.62 | 1.000 | -0.258 |
| SoAS_8 | 316 | 5.37 | 1.75 | -1.056 | -0.058 |
| SoAS_9 | 316 | 5.79 | 1.50 | -1.557 | 1.762 |
| SoAS_10 | 316 | 1.82 | 1.33 | 1.966 | 3.371 |
| SoAS_11 | 316 | 1.68 | 1.31 | 2.174 | 3.995 |
| SoAS_12 | 316 | 5.66 | 1.53 | -1.364 | 1.116 |
| SoAS_13 | 316 | 5.93 | 1.43 | -1.779 | 2.780 |

SoAS_# = Sense of Agency Scale Item

**Supplementary Table 3. Corrected item-total correlations and Cronbach’s alpha if item deleted for SoAS-TR items.**

| **Subscale** | **Item** | **Corrected item-total correlation** |  | **α if item deleted** |
| --- | --- | --- | --- | --- |
| SoPA | SoAS_1 | 0.441 |  | 0.808 |
|  | SoAS_4 | 0.461 |  | 0.800 |
|  | SoAS_8 | 0.621 |  | 0.766 |
|  | SoAS_9 | 0.677 |  | 0.754 |
|  | SoAS_12 | 0.685 |  | 0.751 |
|  | SoAS_13 | 0.542 |  | 0.784 |
| SoNA | SoAS_2 | 0.440 |  | 0.750 |
|  | SoAS_3 | 0.499 |  | 0.740 |
|  | SoAS_5 | 0.514 |  | 0.735 |
|  | SoAS_6 | 0.508 |  | 0.737 |
|  | SoAS_7 | 0.522 |  | 0.733 |
|  | SoAS_10 | 0.481 |  | 0.743 |
|  | SoAS_11 | 0.474 |  | 0.744 |

SoAS_# = Sense of Agency Scale Item; SoNA = Sense of Negative Agency; SoPA = Sense of Positive Agency
